# Association between serum anion gap and risk of in-hospital mortality in patients with acute heart failure

**DOI:** 10.1038/s41598-024-55658-6

**Published:** 2024-02-28

**Authors:** Zilun Huang, Shanjie Wang, Shuang Yang

**Affiliations:** 1 Department of Cardiology, Second Affiliated Hospital of Harbin Medical University, 246 Xuefu Road, Nangang District, Harbin, 150086 China; 2 The Key Laboratory of Myocardial Ischemia, Chinese Ministry of Education, Harbin, China

**Keywords:** Acute heart failure, Serum anion gap, Mortality, MIMIC-IV database, Critically ill, Cardiology, Heart failure

## Abstract

A high serum anion gap (AG) at the time of patient admission can lead to the deterioration or even death; data are lacking for patients who suffer acute heart failure (AHF). The present study aimed at exploring the impact of serum AG (SAG) levels on the in-hospital mortality in AHF patients. The study conducted retrospective analysis on the data from the medical information mart for intensive care (MIMIC-IV) database in severe AHF cases. Serum AG, age, sex, concomitant diseases and laboratory tests were collected from patients at admission. Multivariate Cox proportional hazard regression model together with Kaplan Meier (K–M) survival curve served for analyzing the relationship of serum AG with the hospital all-cause mortality (ACM). In addition, subgroup analysis assisted in assessing the concordance. Data from 2774 AHF patients were collected in the study. The hospital ACM rate was 9.2% (254/2774). After correcting potential confounders, multivariate analysis compared the high serum AG level (≥ 16 mmol/L) and the low serum AG level (< 16 mmol/L) (hazard ratio (HR): 1.89 [95% CI 1.42–2.51]). In a similar way, K–M survival curve indicated that hospital survival was lower in patients with high serum, suggesting that high serum AG level could lead to poor AHF prognosis. In patients with AHF, high serum AG level could increase the hospital ACM.

## Introduction

Acute heart failure (AHF) is an acute or aggravate abnormal left ventricular dysfunction due to reduced cardiac load, increased myocardial contraction force, acute cardiac output decrease, pulmonary pressure, and elevated peripheral circulation resistance, leading to acute pulmonary congestion and development of pulmonary congestion and edema. A source of organ underperfusion and shock is the clinical syndrome, with left heart failure being the most common. AHF can be acutely exacerbated or suddenly outbreak on the basis of the original chronic heart failure. A majority of patients develop organic cardiovascular disease prior to the onset of the disease, manifested as systolic or diastolic heart failure^1^. Acidosis can independently predict the long-term prognosis in AHF, and pH helps stratify risk. Nevertheless, the predictive values for other laboratory parameters reflecting the imbalance between acid and base in AHF, such as serum AG, require further experimental demonstration. Serum AG is the undetermined difference between anions and cations and is readily available as a laboratory parameter. Anion gap (AG) was defined as the difference between unmeasured anions and unmeasured cations, the calculated formula was derived from the concentrations of three commonly measured ions (Na, Cl +  − , HCO − 3)^2^. According to some recent studies, an increased serum AG can largely lead to poor prognosis in various diseases (acute pancreatitis, cerebral stroke, renal injury, trauma, coronary artery disease, etc.)^3–10^. Nevertheless, it remains unclear if serum AG can serve for predicting mortality in severe AHF cases. Hence, the study aimed at investigating the relationship between serum AF level and in-hospital mortality.

## Methods

### Data source

The Medical Information Mart for Intensive Care (MIMIC-IV) (version 2), a large public access database, served for the retrospective cohort study.

The Massachusetts Institute of Technology and the Institutional Review Board of Beth Israel Deaconess Medical Center (BIDMC, Boston, MA, USA) has approved the usage of the database. And all patients admitted to the BIDMC during the years from 2008 to 2019. Zilun Huang, as one of authors of the study, completed the National Institutes of Health's web-based course “Protecting Human Research Participants” (Record ID: 50,160,408) and was granted access to the database for data extraction. Data de-identification was performed for protecting patients’ privacy. Hence, the ethical committee of the Beth Israel Deaconess Medical Center waived patients’ informed consent. The study was described conforming to the Strengthening the Reporting of Observational studies in Epidemiology (STROBE) statement and following the Declaration of Helsinki.

### Population selection

**Figure 1:**
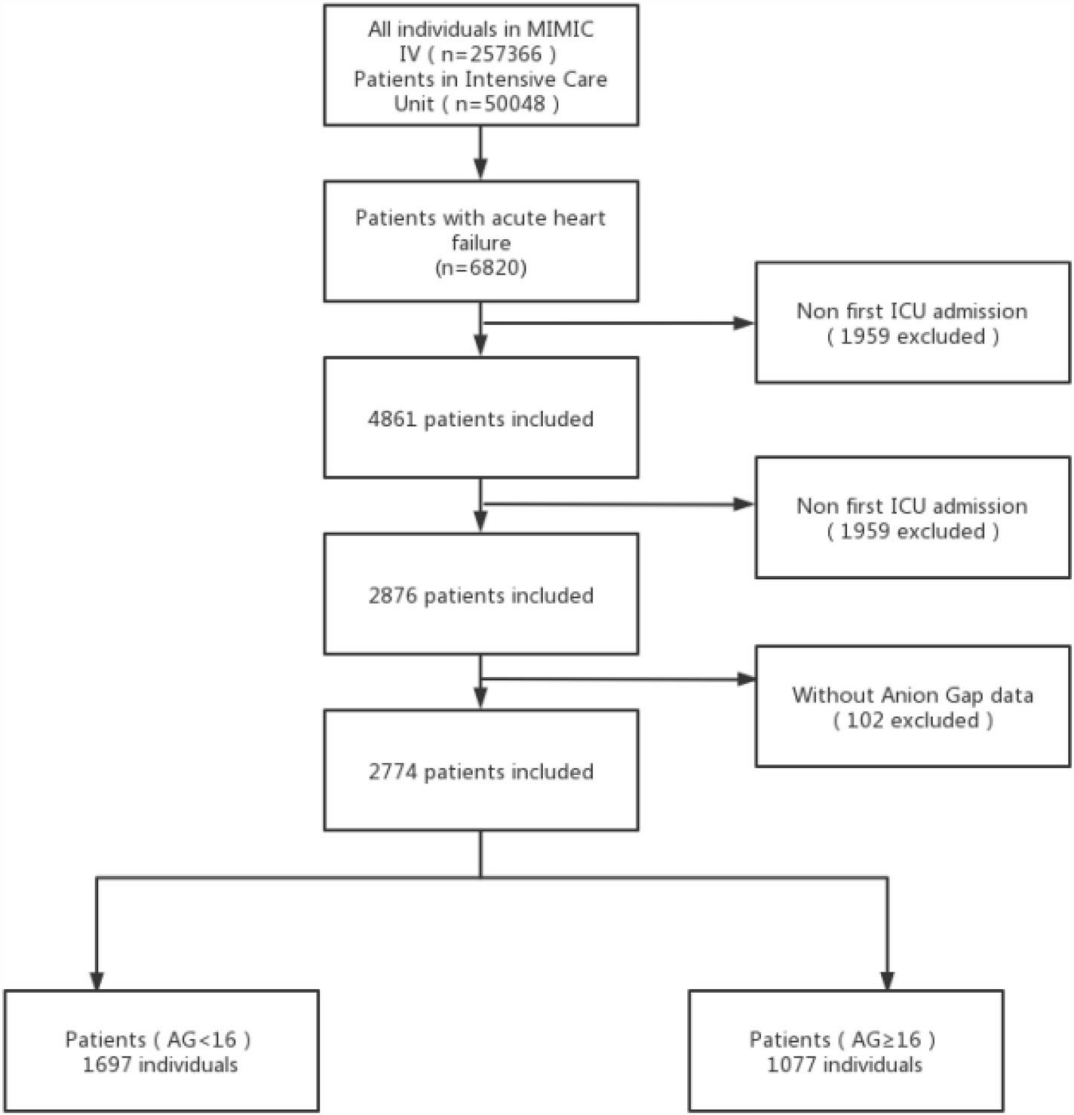
Selection of study population from MIMIC-IV database.

We classified adult patients diagnosed with AHF when they were admitted to hospital according to the International Classification of Diseases version 9 and 10 diagnosis codes (“42,821”, “42,831”, “42,841”, “I5021”, “I5031″, ”I50,811″) in the MIMIC-IV database. Exclusion criteria were: (I) not the first hospitalization; (II) not first ICU (III) Patients without serum AG level; Thus, the study only included 2774 patients (Fig. 1). Our team extracted the first serum AG value of patients after they were admitted as the variable of interest and the primary exposure factor. Structured Query Language (SQL) with PostgreSQL served for extracting all variables from the MIMIC-IV database: demographic variables (age, and sex); vital signs (systolic blood pressure (SBP), diastolic blood pressure (DBP), mean blood pressure (MBP), heart rate, respiratory rate (RR), and percutaneous oxygen saturation (SpO_2_); comorbidities (myocardial infarct (MI), diabetes, peripheral vascular disease (PVD), chronic pulmonary disease (CPD), cerebrovascular disease (CVD), renal diseases, liver diseases, and malignancy cancer) were used for related analysis using the recorded ICD-9 and ICD-10 codes in the database; laboratory variables (WBC count, platelet count, hemoglobin, glucose, sodium, potassium, chloride, creatinine, BUN, and bicarbonate) were acquired at the first test at admission.

### Outcomes

The primary endpoint was the hospital all-cause mortality (ACM), and the definition was based on patients’ survival status when they were discharged from hospitals.

### Statistical analysis

Continuous variables were in the form of means ± standard deviation (SD) or median interquartile ranges (IQR). Categorical variables were in the form of percentages. Kruskal–Wallis test or Mann–Whitney U test assisted in comparing group with low serum AG (AG < 16 mmol/L) and group with high serum AG (AG ≥ 16 mmol/L) statistically. Restricted cubic spline analysis explained that the serum AG level did not present a linear relationship with the hospital ACM in AHF patients. Multivariate Cox proportional risk models were adopted for evaluating above relationship. We selected baseline variables that were considered to present a clinical relevance or to have > 10% change in the effect estimates as confounders. In Model I, age and gender in covariate group were adjusted, and besides these covariates, we also considered SBP, DBP, respiratory rate, heart rate, SPO_2_, diabetes, MI, renal disease, CPD, PVD, CVD, creatinine, malignant cancer, liver disease, WBC, glucose, and BUN in Model II. The K–M curve served for visualizing above relationships. In AHF patients, high AG levels are related to the in-hospital mortality. AG can predict AHF patients’ in-hospital mortality and helps refine risk stratification. We expressed results as HR with a 95% CI *P* < 0.05 reported statistical significance. The statistical software packages R and SPSS served for all analyses.

### Ethics approval and consent to participate

Beth Israel Deaconess Medical Center took charge of examining and approving the studies that involved human participants. Data de-identification was conducted for protecting patient privacy; hence, the Ethical Committee of the Beth Israel Deaconess Medical Center waived patients’ informed consent.

## Results

### Baseline characteristics of subjects

The study selected 2,774 AHF patients, with 1,460 men and 1,314 women (age range: 59–79 years old; average age: 70 years old). The serum AG level-based distribution of patients’ baseline population characteristics are described in Table 1.

**Table 1:** .

Characteristics	Serum anion gap (mmol/L)			p**-**value
	Total	Serum anion gap(< 16)	Serum anion gap(≥ 16)	
	(*n* = 2774)	(*n*** = **1697)	(*n*** = **1077)	
Age, years	70 (59–79)	70 (58–81)	69 (58–79)	0.052
Sex, n (%)				0.594
Male	1460 (52.6%)	900 (53%)	560 (52%)	
Female	1314 (47.4%)	797 (47%)	517 (48%)	
SBP, mmHg, (IQR)	113.9 (105.2–126.4)	113.8 (105.7–125.8)	113.9 (104.4–128.1)	0.664
DBP, mmHg, (IQR)	62.1 (55.0–70.5)	60.8 (54.1–69.4)	63.9 (56.5–71.7)	< 0.001
MBP, mmHg, (IQR)	76.4 (70.1–84.6)	75.8 (69.6–83.4)	77.3 (71.2–86.3)	< 0.001
HR, beats/min, (IQR)	84.8 (75.2–97.0)	83.4 (74.4–94.1)	88.0 (76.5–101.9)	< 0.001
RR, beats/min, (IQR)	19.8 (17.4–22.8)	19.3 (17.1–21.9)	20.8 (19.3–23.9)	< 0.001
SpO2, (IQR)	96.6 (95.2–98.0)	96.7 (95.3–98.1)	96.5 (95.1–97.9)	0.031
Hospital length of stay, days	8.69 (4.99–144.6)	8.60 (5.11–13.94)	8.82 (4.77–15.48)	0.797
Hospital mortality, *n* (%)	254 (9.2%)	103 (6.1%)	151 (14%)	< 0.001
Comorbidities, n (%)				
Myocardial infarct	847 (30.5%)	471 (27.7%)	376 (34.9%)	< 0.001
Diabetes	986 (35.5%)	558 (32.8%)	428 (39.6%)	< 0.001
Peripheral vascular disease	331 (12.1%)	199 (11.7%)	132 (12.8%)	0.434
Chronic pulmonary disease	779 (28.0%)	497 (29.2%)	282 (26.1%)	0.076
Cerebrovascular disease	290 (10.5%)	168 (9.8%)	122 (11.9%)	0.004
Renal disease	780 (28.1%)	359 (21.1%)	421 (39.0%)	< 0.001
Liver disease	231 (8.2%)	129 (7.5%)	102 (9.4%)	0.084
Malignant cancer	320 (11.5%)	191 (11.2%)	129 (11.9%)	0.562
Laboratory tests				
WBC (109/L)	11.3 (8.2–15.1)	10.8 (7.8–14.3)	12.2 (8.9–16.2)	< 0.001
Hemoglobin (g/dL)	10.8 (9.4–12.4)	10.6 (9.3–12.3)	11.1 (9.5–12.6)	< 0.001
Platelet (109/L)	202.0 (149.5–261.3)	193.0 (145.5–249.5)	212.5 (157.5–282.0)	< 0.001
Glucose(mmol/L)	134.0 (131.0–171.5)	128.0 (111.0–158.5)	149.0 (120.0–198.0)	< 0.001
Sodium (mmol/L)	138.0 (135.5–140.5)	138.5 (136.0–140.5)	138.0 (135.0–140.0)	< 0.001
Potassium (mmol/L)	4.3 (3.9–4.7)	4.2 (3.9–4.5)	4.3 (3.9–4.9)	< 0.001
Chloride (mmol/L)	103.5 (99.5–106.5)	104.0 (101.0–107.5)	101.5 (97.5–105.0)	< 0.001
BUN (mg/dL)	23.5 (16.5–37.0)	20.5 (15.5–29.3)	32.0 (20.5–52.5)	< 0.001
Creatinine (mg/dL)	1.2 (0.9–1.7)	1.0 (0.8–1.3)	1.6 (1.1–2.8)	< 0.001
Bicarbonate (mmol/L)	23.0 (20.5–26.0)	24.0 (22.0–26.5)	21.0 (18.5–24.0)	< 0.001

SBP, systolic blood pressure; DBP, diastolic blood pressure; MBP, mean blood pressure; RR, respiratory rate; HR, heart rate; SpO2, percutaneous oxygen saturation;WBC, white blood cell; BUN, blood urea nitrogen.

### Association of serum AG and ACM in AHF patients

**Figure 2:**
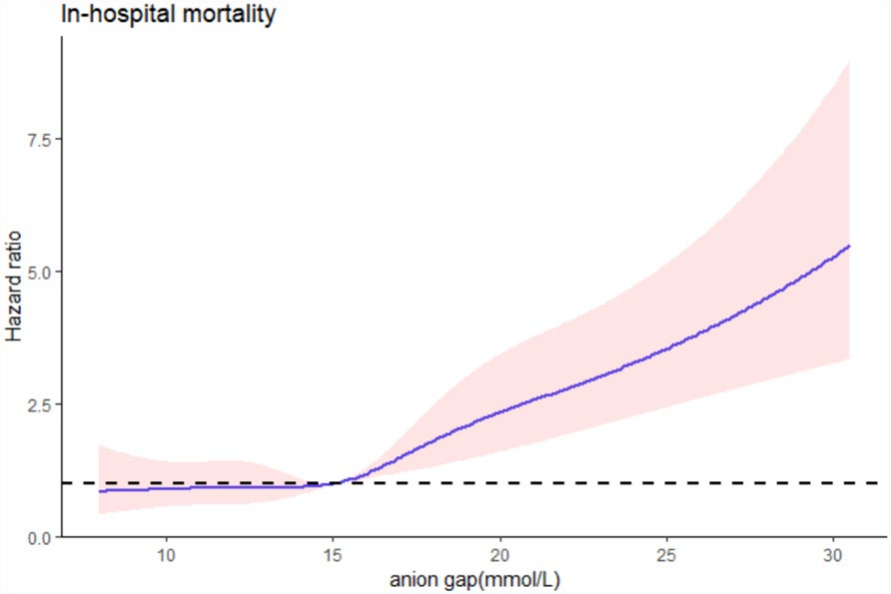
Cubic spline plot of the relation between anion gap and risk of inpatient mortality. The model is fitted using restricted cubic splines with four knots in the generalized additive model. Shaded areas around the curves depict 95% confidence intervals.

**Figure 3:**
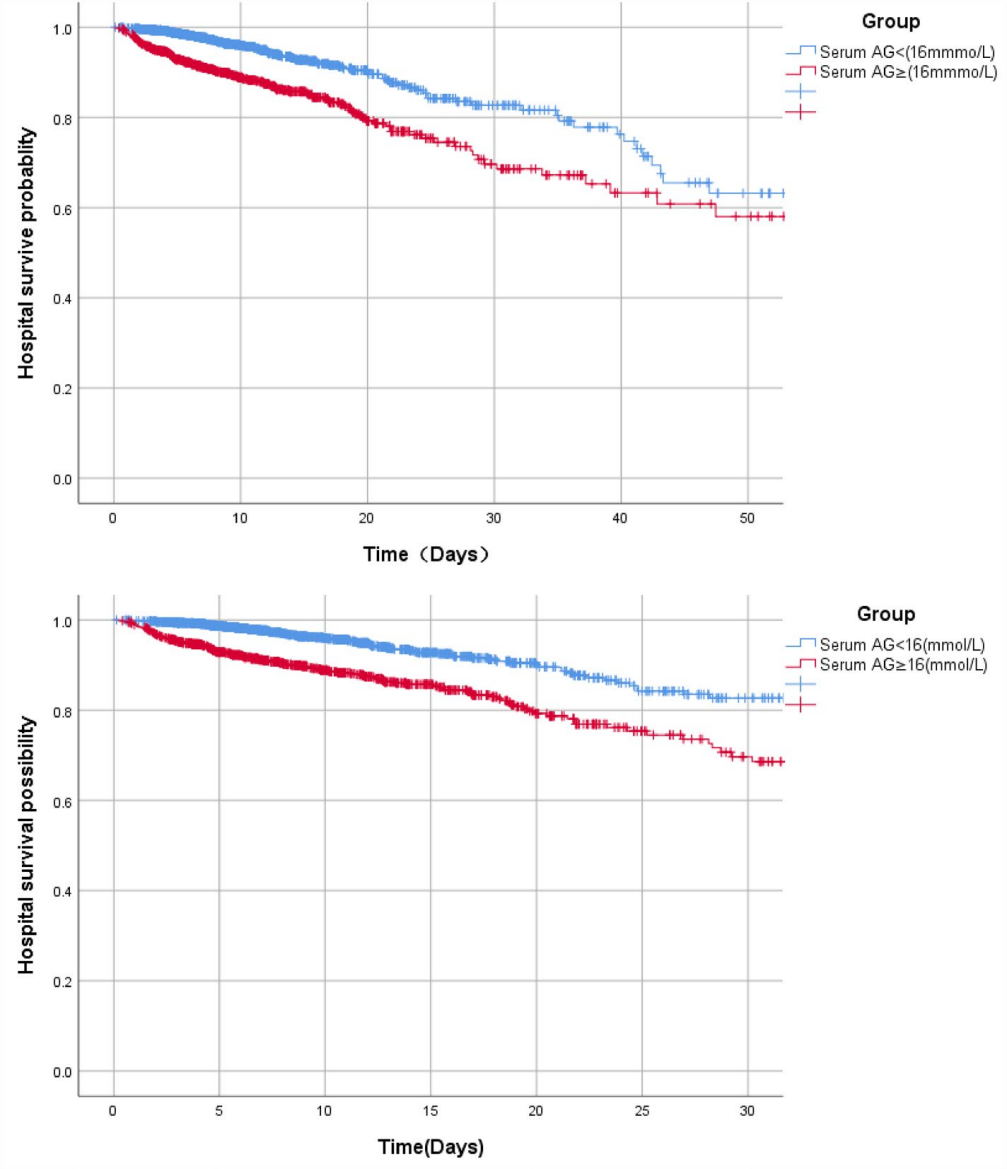
Kaplan–Meier survival curves for critically ill patients with AHF based on serum Anion Gap. x-Axis: survival time (days). y-Axis: survival probability.

According to restricted cubic spline analysis, serum AG was non-linearly associated with the hospital mortality in AHF patients. Serum AG level (< 16 mmol/L) did not present a clear relevance to the hospital ACM of AHF patients. ACM increased with the serum AG levels (≥ 16 mmol/L) (Fig. 2). Cox proportional hazards models served for the adjusted and unadjusted analyses regarding the serum AG level and the ACM in AHF patients (Table 2). AHF patients’ hospital ACM elevated with a per 1-unit elevation in serum AG. Serum AG level served as a categorical variable for the hospital ACM, where the level less than 16 mmol/L was treated as a control. In the crude model, elevated serum AG level led to higher hospital ACM (HR 2.27 [95% CI 1.77–2.93]). In Model I, the adjusted age and gender, as well as high serum AG level led to higher hospital ACM (HR 2.46 [95% CI 1.90–3.17]). Besides, in Model II, adjusted age, SBP, DBP, RR, heart rate, SPO2, diabetes, MI, renal disease, CPD, PVD, CVD, malignant cancer, Liver disease, WBC, glucose, BUN, creatinine, the higher serum AG, exhibited an obvious relevance to higher hospital ACM (HR 1.89 [95% CI 1.42–2.51]), and group with low serum AG level was taken as a control. Besides, according to the KM survival curve, patients whose serum AG level was ≥ 16 mmol/L after they were admitted had lower survival rate (*P* < 0.001) (Fig. 3).

**Table 2:** Multivariable cox regression models evaluating the association between serum Anion Gap and hospital all-cause mortality.

Variable	Crude		Model I		Model II	
	HR (95%CI)	*p*-value	HR (95%CI)	*p*-value	HR (95%CI)	*p*-value
Serum AG < 16 mmol/L	1 (Ref)		1 (Ref)		1 (Ref)	
Serum AG ≥ 16 mmol/L	2.27 (1.77–2.93)	< 0.001	2.46 (1.90–3.17)	< 0.001	1.89 (1.42–2.51)	< 0.001

Crude model: adjusted for none.

Model I: adjusted for age, sex.

Model II: adjusted according to Model I + SBP, DBP, respiratory rate, heart rate, SPO2, diabetes, myocardial infarct, renal disease, chronic pulmonary disease, peripheral vascular disease, cerebrovascular disease, malignant cancer, Liver disease, WBC, glucose, BUN, creatinine.

### Subgroup analyses

**Figure 4:**
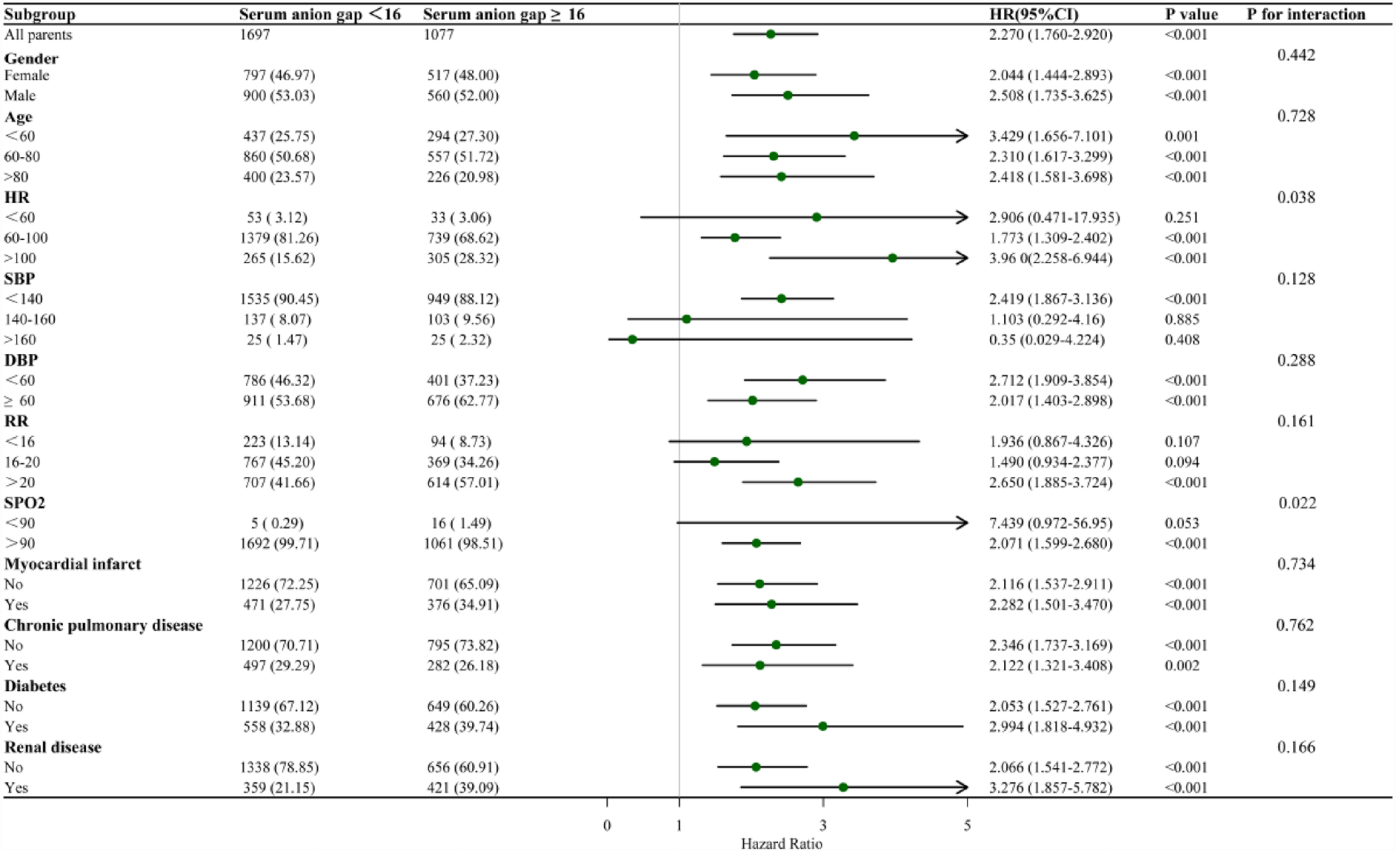
Subgroup analyses were performed to evaluate the association between high serum AG levels and hospital all-cause mortality.

Subgroup analyses served for assessing the relation of high serum AG level to hospital ACM (Fig. 4). Subgroup analysis followed the strata: age, gender, SBP, DBP, HR, RR, SPO_2_, and major comorbidities, such as MI, CPD, diabetes, and renal disease. In most subgroups, results were consistent with the primary analysis for each subgroup of the population. Age, gender, HR ≥ 60, SBP < 140, DBP, SPO2 > 90, LODS, MI, CPD, diabetes, and renal disease were remarkably changed.

## Discussion

AHF is an acute cardiovascular disease characterized by high mortality. Unfortunately, there are no objective indicators predicting AFH patients’ long-term prognosis. Clinical laboratory tests, which included blood gas analysis and biochemical tests, commonly assisted in confirming patient status, but few studies have been performed on the impact of these indicators on AHF patients’ prognosis. Therefore, the search for an easier and valid laboratory parameter for predicting AHF patients’ prognosis is of large value and importance for the clinical management.

In the study, subjects who had serum AG level ≥ 16 mmol/L) had lower hospital admission survival, shorter survival times, and higher serum AG levels at admission increased in-hospital mortality in AHF patients. To be specific, patients whose serum AG levels were high had a 1.89-fold higher in-hospital ACM (≥ 16 mmol/L) relative to patients whose serum AG levels were low (< 16 mmol/L), respectively.

Serum AG is elevated due to excess acid production or decreased anion excretion in general^11^. Clinically, serum AG is easily calculated and routinely measured in hospitalized patients^12^. Therefore, a lot of studies have examined the impact of serum AG levels on the clinical prognosis and mortality of severe AHF cases. In studies that adopted the data in the MIMIC database, higher serum AG level led to elevated ACM in AHF patients^13^. After the adjustment of confounders, patients whose serum AG level was high (AG ≥ 16 mmol/L) had a 1.89-fold higher ACM in the ICU and hospital, relative to patients whose serum AG level was low. In addition, according to the systematic review and meta-analysis conducted recently, serum AG is reliable data for the assessment of critically ill cases’ prognosis, particularly in regions with underdeveloped medical resources^14^. Besides, studies have shown a number of common co-morbidities in patients with AHF, such as diabetes, PVD, chronic lung disease, kidney disease and CVD.

In clinical practice, reductions of SAG are uncommon, possibly explaining the reductions in untested anions (hypoalbuminemia) or laboratory-induced errors^15^. Albumin irreplaceably impacts the the physiological process, e.g. maintenance of colloid osmotic pressure inside and outside blood vessels and the integrity of microvessels, binding of receptors and ligands, and assistance in substance transport, antioxidant and enzymatic activity of the organism^16^. Low levels of serum albumin are capable of aggravating circulatory congestion and enhancing oxidative stress, inflammatory response as well as infection sensitivity, thereby worsening heart failure patients’ prognosis^17^. In patients with AHF, hypoproteinemia partially increases the hospital mortality and can independently predict the long-term mortality^18^. SAG will increase in general cases. According to previous studies, 62% of AG increase is resulted from serum lactate and ketone body accumulation^19^. In AHF, the heart can not effectively pump blood, which results in reduced tissue perfusion and cellular hypoxia^20^. In anaerobic environment, glucose glycolysis occurs to produce lactic acid, primary explaining the increase in SAG in AHF patients^21^. Besides, sympathetic excitation in AHF will result in a large amount of lactic acid production^22^. Lactic acid generation and excretion are normally balanced, with the liver and kidney playing a major role in this process. However, liver and renal disorders are frequently present in AHF patients, as a result, lactic acid and SAG accumulate in the body.

The study has some limitations. First, although a multivariable model was used for controlling for the effect of confounding factors on outcome variables, we did not take into account some unknown or important risk factors. Second, patients’ SAG concentrations were used only at admission to assess their association with ACM. Dynamic monitoring of SAG during clinical work would exhibit larger predictive value. Third, albumin correction was not performed for SAG due to the lack of albumin records, despite of the impact of hypoalbuminemia on SAG concentrations as reported in some studies. At last, we failed to compare the predictive accuracy and efficiency of NT-proBNP and SAG regarding the poor outcomes in severe AHF cases, so exclusion bias may exist.

In conclusion, this retrospective observational study revealed the relevance of high serum AG levels to ACM in AHF patients. It is required to conduct further prospective clinical studies that involve larger sample sizes for better assessing the causal relationship of high serum AG levels with nosocomial ACM.

## Data Availability

The data in the study was provided by the Medical Information Mart for Intensive Care IV (MIMIC-IV), and the following licenses/restrictions apply: To obtain access to these files, you are required to be a credentialed user, finish necessary training as well as sign the project data use agreement. The datasets used and analysed during the current study available from the corresponding author on reasonable request.
